# Environment or economy? Individual and country-level predictors: an international comparative analysis

**DOI:** 10.3389/fpsyg.2026.1916215

**Published:** 2026-08-14

**Authors:** Fatih Kaya

**Affiliations:** Department of Social Studies Education, Faculty of Education, İnönü University, Malatya, Türkiye

**Keywords:** education, environmental priority, environment-economy trade-off, post-materialism, World Values Survey

## Abstract

This study aims to examine the individual and country-level determinants of individuals’ preferences between environmental protection and economic growth within an international comparative framework. Conducted across 66 countries as part of the 7th Wave of the World Values Survey (WVS), the study is based on a sample of 86,532 individuals who provided valid responses to item Q111. The variables identified were, at the individual level, education, gender, age, income and place of residence; and at the country level, GDP per capita, duration of compulsory education, expenditure on education and CO₂ emissions. The study’s data were analyzed using weighted logistic regression models incorporating country fixed effects. The findings of the study indicate that the strongest individual factor increasing an individual’s likelihood of prioritizing the environment is level of education; women exhibit higher environmental preferences than men, and younger people exhibit higher environmental preferences than older people. Individual income level and urban residence, on the other hand, are negatively associated with environmental prioritization. At the country level, per capita GDP, education expenditure and the duration of compulsory education are positive predictors of environmental prioritization, while CO₂ emissions are a negative predictor.

## 1 Introduction

Global environmental threats such as the climate crisis, biodiversity loss and environmental pollution are among the most significant challenges of the 21st century (Shin et al., 2022). To tackle these challenges, countries must prioritize environmental issues at both the public and individual levels. Indeed, the international literature demonstrates that environmental priorities vary significantly between countries and individuals (Dunlap and York, 2008; Franzen and Vogl, 2013). Understanding the individual and institutional reasons underlying this variation is of critical importance in terms of education systems and environmental policies.

Although education is one of the most decisive factors in shaping environmental attitudes and values (Arcury and Christianson, 1993; Chanda, 1999; Gifford and Nilsson, 2014), in comparative studies it is generally used as an indicator of socio-economic status; its individual and structural dimensions are not examined. This gap represents a significant lacuna in comparative environmental sociology and educational research.

Inglehart and Welzel’s (2005) theory of post-materialism posits that, as economic security increases, individuals turn towards self-expression values-such as environmental protection-beyond material concerns. While this theory continues to serve as the fundamental theoretical framework for environmental attitude research, Dunlap and York (2008) provide evidence that environmental concern persists strongly in many societies, regardless of the level of economic development. This debate necessitates multi-level and multi-variable approaches that go beyond single-variable and linear explanatory models. The 7th wave of the World Values Survey (WVS) (Haerpfer et al., 2024), with data collected from 97,220 individuals across 66 countries, constitutes one of the most comprehensive and up-to-date datasets contributing to this debate. However, the number of international studies that simultaneously model both individual and country-level variables is quite limited, and few of these consider country fixed effects and contextual variables together. The effect of the education system on individuals, in particular, has not been sufficiently examined.

At this stage, the research focuses on the following questions: When sociodemographic variables are controlled for, does individual educational attainment significantly predict environmental priorities? How are individual variables such as gender, age, income and place of residence related to environmental priorities? Do country-level structural variables-such as GDP per capita, duration of compulsory education, expenditure on education and CO₂ emissions-predict environmental priorities independently of individual factors?

The aim of this study is to examine, within an international comparative framework, how the priority that people give to environmental protection over economic growth is shaped at the individual and country levels. The study makes two main contributions to the literature. Theoretically, it treats education not only as an individual attribute but also as a country-level one, and carries the post-materialism debate to the institutional level. In practical terms, the findings show under which individual and country-level conditions environmental priorities are strengthened, and in this way contribute to education and environmental policy.

## 2 Theoretical framework

### 2.1 Post-materialism and modernization

One of the most widespread theoretical frameworks for explaining the distribution of environmental priorities amongst individuals and countries is undoubtedly based on the post-materialism thesis. Inglehart (1977) argues that, in the process of social transformation he termed the ‘silent revolution’, under conditions of economic prosperity, individuals move beyond material concerns to embrace post-materialist values such as quality of life, individual freedom and environmental protection. This framework posits that as the pressures of insecurity and hunger diminish-that is, as generations grow up in an environment of economic and physical security-they are drawn more strongly towards values such as environmental concern. Indeed, Inglehart (1995) empirically supported this framework through a comparative analysis covering 43 countries. Inglehart (1997), meanwhile, examined the argument within a broader framework encompassing institutional structures, the education system and the development of civil society, viewed through the lens of modernization and post-modernization processes. Inglehart and Welzel (2005) outlined the link between survival values and self-expression values within a systematic framework, thereby demonstrating the strong relationship between environmental awareness and self-expression values.

The universal validity of this thesis has been called into question over time. Indeed, Brechin (1999) demonstrated that environmental concern is not limited solely to high-income countries, while Dunlap and Mertig (1995, 1997) showed that environmental concern in developing countries can reach levels that the post-materialism thesis could not have predicted. Similarly, Dunlap and York (2008), using findings derived from data across multiple countries, demonstrated that environmental concern is not a phenomenon exclusive to high-welfare and developed countries; on the contrary, it is also clearly evident in developing countries. These findings indicate that economic prosperity alone is not a sufficient determinant of environmental concern and that this concern is of a global nature. In summary, these findings suggest that not only individual levels of prosperity but also factors such as national economic development, the education system and environmental pressures may play an independent and decisive role in shaping environmental priorities. This study aims to contribute to this debate by simultaneously examining both the post-materialist hypothesis and alternative explanatory frameworks that go beyond this hypothesis, using data from 66 countries collected as part of the 7th wave of the World Values Survey (WVS) (Haerpfer et al., 2024).

### 2.2 Individual predictors of environmental prioritization

Factors influencing individual environmental attitudes have been extensively examined in the comparative social sciences literature. Gifford and Nilsson (2014) systematically examined 18 personal and social factors influencing environmental behavior and identified education, alongside knowledge, as one of the most significant personal predictors of pro-environmental attitudes and behavior. Franzen and Vogl (2013), drawing on findings from the 33-country ISSP, demonstrated that education is positively associated with environmental concern and that environmental concern is also high in developed countries. Building on this, modelling the individual education variable alongside country-level education indicators enables us to distinguish between the individual and structural dimensions of the education-environment relationship.

Gender is one of the key explanatory variables associated with environmental attitudes in the literature. Research (Zelezny et al., 2000; Desrochers et al., 2019) has shown that women exhibit higher levels of environmental sensitivity than men. This difference in favor of women is often explained from the perspective of gender socialization and the ethics of care. This perspective also highlights women’s relational responsibility and protection-oriented understanding (Gilligan, 1982; Noddings, 1984). On the other hand, Xiao and McCright (2015) found that institutional trust does not account for this gender difference.

Research on the age variable presents a more inconsistent picture. Indeed, while some studies report that young people have higher levels of environmental concern, it is also noted that this may be linked to a generational effect (Gifford and Nilsson, 2014). Although individual income is positively associated with environmental concern, differences in GDP between countries demonstrate a much stronger contextual influence (Franzen and Vogl, 2013). Similarly, urban–rural differences are also associated with environmental attitudes; however, this distinction is multidimensional in nature (Gifford and Nilsson, 2014).

There is comparative evidence to suggest that the relationship between individual income and environmental priorities is not one-way, that this relationship may vary from country to country, and that individual and national income indicators may operate through different mechanisms (Brechin, 1999; Marquart-Pyatt, 2008).

### 2.3 Country-level factors and a comparative perspective

Comparative studies emphasize that explaining environmental concern solely through individual characteristics is insufficient and that the national context is also significant in this regard. Indeed, Marquart-Pyatt (2008) demonstrated that models explaining environmental concern are largely similar across industrialized countries. In his study covering 50 countries, Gelissen (2007) demonstrates that environmental priorities are linked to country-level factors such as patterns of economic development and cultural values. These findings emphasize that environmental priorities are not merely a function of economic prosperity, but also of political institutions and public engagement.

Inglehart and Welzel’s (2005) world values map reveals systematic differences between cultural regions by visualizing countries’ positions on the traditional-secular-rational and survival-self-expression axes. This map presents both the value judgements and environmental sensitivities of eight major cultural regions-Protestant Europe, Catholic Europe, Orthodox Europe, Confucian regions, Latin America, South Asia, the Islamic world and Africa-using an inter-regional approach. However, cultural region classifications carry the risk of homogenizing intra-regional variation; significant differences are observed between countries within the same cultural bloc in terms of economic development, education systems and environmental pressures. For this reason, it is necessary to control for country-level variables when assessing the influence of cultural regions. Accordingly, cultural region is used in this study as a descriptive classification rather than as a categorical predictor in the main models. The analytical focus is on theoretically specified and substantively interpretable structural indicators-economic development, education-system characteristics, and environmental pressure-which may help explain cross-national variation without assuming homogeneity within broad cultural regions.

Country-level variables include GDP per capita, duration of compulsory education, expenditure on education and CO₂ emission intensity. These are country-level variables; in other words, they help to account for the differences between countries that individual variables cannot explain on their own. At this point, modelling individual and country-level variables together allows for a more accurate interpretation of contextual effects by distinguishing between differences between individuals and between countries (Snijders and Bosker, 2012). This study is similar to previous research that has opted for a multilevel regression model using WVS data. In their multi-level study based on ISSP data, Franzen and Meyer (2010) examined the effect of cross-national GDP differences on environmental attitudes and concluded that environmental concern is higher in wealthier countries. Diekmann and Franzen (1999), while demonstrating that national wealth is a significant explanatory factor, emphasize the need to assess environmental concern within a broader framework.

While one might expect environmental priorities to be higher in countries with high CO₂ emissions-which are among the country-level variables-research points to the opposite being the case. In line with the findings of Dunlap and York (2008), this study found that CO₂ emissions are negatively correlated with environmental priorities. This result can be summarized as follows: in industrialized, high-emission countries, the desire for economic growth overshadows environmental concerns. From this perspective, modelling the relationship between CO₂ emission intensity and environmental concern within a national context can be regarded as a model rarely undertaken in the literature.

The impact of countries’ education systems on environmental attitudes is also attracting increasing attention in comparative studies. The duration of compulsory education and the amount of public resources allocated to education are regarded as structural factors determining the quality of the individual learning process. A critical question that has emerged is whether these structural indicators can predict environmental attitudes independently of the impact of individual education (Marquart-Pyatt, 2008; Snijders and Bosker, 2012). This study adopts an integrated approach that simultaneously models individual and country-level variables and incorporates country-specific fixed effects, with the aim of addressing this analytical gap (Dunlap and York, 2008; Haerpfer et al., 2024).

Based on this theoretical framework and the relevant empirical literature, the nine hypotheses of the study have been formulated as follows. The individual-level hypotheses (H1-H5) test the independent effects of education, gender, age, income and place of residence on environmental priorities; the country-level hypotheses (H6-H9) test the structural effects of GDP, duration of compulsory education, education expenditure and CO₂ emissions:

*H1*: As an individual variable, educational attainment is primarily related to environmental priority.

*H2*: As an individual variable, gender is primarily related to environmental priority.

*H3*: As an individual variable, age is primarily related to environmental priority.

*H4*: As an individual variable, income is primarily related to environmental priority.

*H5*: As an individual variable, place of residence is primarily related to environmental priority.

*H6*: As a country-level variable, GDP per capita is primarily related to environmental priority.

*H7*: As a country-level variable, the duration of compulsory education is primarily related to environmental priority.

*H8*: As a country-level variable, education expenditure is primarily related to environmental priority.

*H9*: As a country-level variable, per capita CO₂ emissions are primarily related to environmental priority.

## 3 Method

### 3.1 Research model

This study is a quantitative, cross-sectional and international comparative study that examines the predictive effect of individual socio-demographic and socio-economic variables, as well as country-level indicators, on environmental-economic priorities.

### 3.2 Sample and data source

The data source for this study is the 7th wave of the World Values Survey (WVS) (Haerpfer et al., 2024). The WVS is an international research program that has been conducted since 1981 and systematically measures human values, beliefs and attitudes through multi-stage stratified sampling. The data set used (Version 6.0.0) contains responses from 97,220 individuals aged 18 and over across 66 countries.

The total sample for Wave 7 of the WVS comprises 97,220 individuals. Of the 88,515 individuals who provided valid responses to item Q111, 86,532 formed the core analysis sample following listwise deletion of individual-level independent variables; responses marked as invalid, missing or ‘both are important’ were subsequently excluded from the analysis. The M3 full model includes 48 countries and 65,864 individuals for which data on country-level variables are available. The harmonic mean sample size per country is 1,337.5. Eighteen countries were excluded from M3 because at least one of the four country-level indicators was unavailable. Most exclusions were associated with missing data on education expenditure. This sample size meets the statistical power requirements necessary for multilevel analysis (Hox et al., 2010; Snijders and Bosker, 2012). Following listwise deletion, the M1 and M2 models were estimated across 65 countries (*n* = 86,532), while the ICC analysis was calculated using dependent variable data from all 66 countries.

Listwise deletion was applied to missing data; as the proportion of missing data in individual variables remained below 5 percent, this approach was deemed acceptable (Graham, 2009). In all models, WVS probability-weighted sampling weights (PWGHT) were used to ensure that the sample better reflected the national population structure (Haerpfer et al., 2024).

### 3.3 Measurements

The dependent variable is the environment-economy preference measured by item Q111: ‘Protecting the environment vs. economic growth and creating jobs.’ In the original World Values Survey (WVS) coding, this item was coded as 1 = environmental protection and 2 = economic growth; for the purposes of this analysis, it has been recoded as 1 = prioritizing the environment and 0 = prioritizing the economy. This coding is suitable for binary logistic regression and is consistent with international comparative studies (Hosmer et al., 2013). The primary independent variable is educational attainment (Q275). This variable is coded according to ISCED 2011, ranging from 0 = no schooling to 8 = doctoral degree (UNESCO Institute for Statistics, 2012). Other individual-level variables include gender (Q260; 1 = female, 0 = male), age (Q262; continuous, years, z-standardized), income (Q288; self-reported, 1–10, z-standardized) and place of residence (HURBRURAL; 1 = urban, 0 = rural). As a self-reported income scale, Q288 reflects respondents’ reported income position rather than a harmonized measure of absolute income or purchasing power across countries.

Country-level variables were obtained from the World Bank (2019) database: (1) GDP per capita (based on purchasing power parity, transformed using the natural logarithm, *z*-standardized), (2) duration of compulsory education (years, *z*-standardized), (3) ratio of education expenditure to GDP (%, *z*-standardized) and (4) per capita CO₂ emissions (tonnes, transformed using the natural logarithm, *z*-standardized). Logarithmic transformations were applied to correct for high skewness and to facilitate interpretation. The distributions and means of the variables used in the study are presented in Table 1.

**Table 1:** Descriptive statistics for the study variables.

**Variable**	***n***	***M***	**SD**	**Range**	**Description**
Q111—Environmental priority (1 = Environment, 0 = Economy)	86,532	—	—	0–1	59.0% Environment; 41.0% Economy
Q275—Education level (ISCED 0–8)	86,532	3.56	2.02	0–8	ISCED 3 mode (25.7%); ISCED 6 (17.6%)
Q260—Gender (1 = Female, 0 = Male)	86,532	—	—	0–1	52.1% Female; 47.9% Male
Q262—Age (years)	86,532	43.0	16.5	16–103	*z*-standardized
Q288—Income (self-reported, 1–10)	86,532	4.92	2.08	1–10	*z*-standardized
HURBRURAL—Place of residence (1 = Urban, 0 = Rural)	86,532	—	—	0–1	67.3% Urban; 32.7% Rural
GDPpercap—GDP per capita (PPP, $)	48 countries	24,262	22,908	2,312–101,376	ln transformation; ln *M* = 9.71, SD = 0.91
compulseduc—Duration of compulsory education (years)	48 countries	10.43	2.56	5–17	*z*-standardized
educationexp—Education expenditure (% of GDP)	48 countries	4.56	1.38	1.50–7.50	*z*-standardized
co2percap—CO₂ emissions per capita (tonnes)	48 countries	4.92	3.99	0.14–15.54	ln transformation; ln M = 1.19, SD = 1.02

WVS = World Values Survey, Wave 7, Version 6.0.0 (Haerpfer et al., 2024). ISCED = International Standard Classification of Education (UNESCO Institute for Statistics, 2012). M = mean; SD = standard deviation; ln = natural logarithm. Country-level n values indicate the number of countries included in the M3 analysis. The reported range values for country-level variables reflect the raw data values for the 48 countries included in the M3 analysis. WVS sampling weights (PWGHT) were used in the analyses. Sources: Haerpfer et al. (2024), UNESCO Institute for Statistics (2012), and World Bank (2019).

### 3.4 Analysis strategy

The clustering of individuals within countries may weaken the assumption of independence in single-level logistic regression and lead to standard errors being underestimated (Rabe-Hesketh and Skrondal, 2006; Raudenbush and Bryk, 2002). This expectation was confirmed by a likelihood ratio (LR) test comparing the model including country fixed effects (M1) with the pooled logit model (M0): χ^2^(67) = 4,941.64, *p* < 0.001. This finding indicates that country-specific unobserved differences constitute a significant source of variance in the dependent variable. Therefore, when the binary categorical nature of the dependent variable and the issue with the independence assumption were considered together, logistic regression with country fixed effects was selected as the most appropriate analytical method (Hosmer et al., 2013).

During the analysis process, an intra-class correlation (ICC) analysis was first carried out. Subsequently, the latent variable approach recommended for binary dependent variables (the π^2^/3 method) was used (Snijders and Bosker, 2012); the value calculated using the formula ICC = σᵤ^2^ / (σᵤ^2^ + π^2^/3) was found to be 0.004 [*F*(65, 86, 466) = 66.10, *p* < 0.001]. Although the ICC is small (0.004), the significant F-statistic and the likelihood-ratio test comparing the country fixed-effects model with the pooled logit model indicate statistically detectable country-specific heterogeneity; country fixed effects were therefore retained to control for unobserved country characteristics. Given the large number of individual observations relative to the number of country indicators, overfitting due solely to the inclusion of country fixed effects is unlikely.

In the study, the models were constructed in four stages: M0, the empty model containing only the constant term; M1, the basic individual model including education, gender and age; M2, the full individual model with all individual variables included; and M3, the full model incorporating both individual and country-level variables. Each model was compared with the previous one using the LR χ^2^ test; model fit was assessed using AIC, BIC and McFadden’s pseudo-*R*^2^ (Hosmer et al., 2013; Luke, 2004). No LR χ^2^ test was performed between M2 and M3. This is because the two models were estimated on different samples (M2: *n* = 86,532; M3: *n* = 65,864). The fixed-effects approach reduces bias in the individual-level coefficients by controlling for unobservable country-specific differences. The low ICC value (0.004) also indicates that the majority of the variance is independent of the country level. In the M3 model, country dummy variables were removed and replaced with country-level continuous variables (lnGDP, CO₂, education expenditure, compulsory education duration). This structure prevents the problem of full multicollinearity and ensures that contextual effects are estimated (Raudenbush and Bryk, 2002). Multicollinearity among the independent variables was tested using VIF; as all values remained below the 10 threshold, no such issue was identified (Hosmer et al., 2013). Thus, the M3 model was established as a model focusing on contextual effects.

In the study, all continuous independent variables were z-standardized (M = 0, SD = 1) and, bearing in mind that a large sample size can render *p*-values significant even for small effect sizes, findings regarding statistical significance were interpreted alongside the OR values. Analyses were conducted in the Google Colab environment using Python 3.12.13 via the statsmodels library (0.14.6; Seabold and Perktold, 2010). The level of statistical significance was set at *α* = 0.05, and effect sizes were reported as odds ratios (OR) and 95% confidence intervals (Hosmer et al., 2013).

*Code Availability*: The Python (version 3.12.13) and statsmodels (version 0.14.6) analysis code used in the estimation of all models (M0, M1, M2, M3), the calculation of descriptive statistics and the creation of tables will be made available in a public code repository (GitHub/Zenodo) upon publication of the article. The WVS Wave 7 data are publicly available and can be downloaded free of charge from www.worldvaluessurvey.org.

## 4 Findings

### 4.1 Distribution of the dependent variable

In the World Values Survey (WVS) Wave 7 sample, 88,515 participants provided valid responses to item Q111. Upon examination of the valid responses, 59.0 percent of participants (*n* = 52,224) prioritized environmental protection over economic growth, while 41.0 percent (*n* = 36,291) prioritized economic growth. This distribution reflects the fact that environmental concern is relatively widespread across the countries covered by the WVS (see Table 2).

**Table 2:** Item Q111: Allocation of priorities between the environment and the economy (*N* = 88,515).

Category	*n*	%	Valid %	Cumulative %
1 — Environment prioritized	52,224	53.7	59.0	59.0
0 — Economy prioritized	36,291	37.3	41.0	100.0
Total	88,515	100.0	100.0	**—**

The system’s missing values include the categories ‘both are important’, ‘do not know’ and ‘no response’. The number of valid responses, *n* = 88,515, was calculated from the raw data.

### 4.2 International comparison

An examination of Table 3 reveals that, out of 66 countries, a total of 52 countries predominantly prioritize environmental protection. In the remaining 14 countries, the economic orientation predominates. At country level, the rates of environmental priority vary across a fairly wide range, from 81.5 percent in Andorra to 36.6 percent in Egypt and Tunisia (see Table 3). At the top of the ranking are Andorra (81.5 percent), Indonesia (77.8 percent) and Bolivia (74.6 percent); at the bottom are Tunisia (36.6 percent), Egypt (36.6 percent) and Lebanon (38.2 percent). When assessed within the framework of the cultural region classification (Inglehart and Welzel, 2005), it is evident that all countries in the Protestant European (8/8), Confucian (8/8) and South Asian (5/5) regions exceed this threshold; by contrast, in the Islamic (9/17) and African (1/4) regions, the majority in question is much more limited.

**Table 3:** Environmental priority rates by country and cultural region (*n* = 66).

Rank	Country	Cultural region	%	*n*	Rank	Country	Cultural region	%	*n*
1	AND	Catholic Europe	81.5	895	34	ECU	Latin America	57.5	1,154
2	IDN	South Asia	77.8	2,976	35	MNG	Orthodox	57.5	1,543
3	BOL	Latin America	74.6	1,978	36	KOR	Confucian	57.5	1,244
4	VNM	Confucian	73.3	1,174	37	HKG	Confucian	56.8	1,959
5	URY	Latin America	72.6	898	38	MEX	Latin America	56.5	1,682
6	NLD	Protestant Europe	72.4	1,734	39	MAR	Islamic	55.2	1,115
6	CHN	Confucian	72.4	2,852	40	CYP	Orthodox	54.9	869
8	PRI	Latin America	72.1	1,028	41	ZWE	Africa	54.1	1,201
9	NZL	Protestant Europe	70.5	828	42	THA	South Asia	54.1	1,438
10	COL	Latin America	70.1	1,501	43	SVK	Catholic Europe	53.8	1,106
11	DEU	Protestant Europe	69.8	1,388	44	MMR	South Asia	51.4	1,199
12	IRN	Islamic	68.5	1,417	45	RUS	Orthodox	51.3	1,568
13	GTM	Latin America	68.5	1,044	46	UKR	Orthodox	51.2	1,136
14	GBR	Protestant Europe	68.4	2,439	47	ARM	Orthodox	51.2	1,147
15	PHL	South Asia	68.1	1,192	48	JOR	Islamic	51.0	1,120
16	AUS	Protestant Europe	67.3	1,769	49	KAZ	Islamic	50.8	1,097
17	KGZ	Islamic	67.1	1,122	50	LBY	Islamic	50.6	1,069
18	BRA	Latin America	66.4	1,441	51	SRB	Orthodox	50.4	894
19	NIC	Latin America	66.2	1,148	52	ARG	Latin America	50.2	835
20	NIR	Protestant Europe	65.6	421	53	CZE	Catholic Europe	48.8	1,120
21	TWN	Confucian	64.4	1,214	54	BGD	Islamic	47.7	1,131
22	MYS	Islamic	63.4	1,250	55	KEN	Africa	46.9	1,214
23	MAC	Confucian	62.9	999	56	ETH	Africa	46.7	1,203
24	UZB	Islamic	62.6	934	57	IRQ	Islamic	44.7	1,159
25	CAN	Protestant Europe	61.2	4,018	58	TJK	Islamic	44.6	1,176
26	SGP	Confucian	60.6	1,844	59	MDV	Islamic	44.3	1,027
27	PER	Latin America	60.0	1,336	60	PAK	Islamic	43.0	1,869
28	IND	South Asia	59.6	1,442	61	NGA	Africa	41.4	1,209
29	JPN	Confucian	59.3	767	62	ROU	Orthodox	41.1	1,118
30	USA	Protestant Europe	59.2	2,306	63	VEN	Latin America	39.5	1,144
31	GRC	Orthodox	59.1	1,059	64	LBN	Islamic	38.2	1,174
32	CHL	Latin America	57.9	929	65	EGY	Islamic	36.6	1,037
33	TUR	Islamic	57.8	2,318	66	TUN	Islamic	36.6	1,094

Environment % = the proportion of participants who prioritize environmental protection over economic growth. The classification of cultural regions is based on Inglehart and Welzel’s (2005) World Values Map. Source: Haerpfer et al. (2024).

### 4.3 Results of logistic regression analysis

#### 4.3.1 Model fit

The fit statistics for the models are presented in Table 4. Model M1 provides a significantly better fit than the null model [χ^2^(67) = 4,941.64, *p* < 0.001]. Model M2 significantly improved model fit compared with M1 [χ^2^(2) = 40.64, *p* < 0.001]; this finding indicates that the inclusion of the income and place of residence variables in the model made a statistically significant contribution. Model M3, which includes all variables, achieved the lowest AIC (88,407.13) and BIC (88,498.09) values.

**Table 4:** Logistic regression model fit statistics.

Model	*n*	K	Log-LL	AIC	BIC	McFadden *R*^2^	LR test
M0 (Null model)	86,532	1	−58,574.24	117,150.49	117,159.86	—	—
M1 (Individual — baseline)	86,532	4	−56,103.42	112,342.84	112,979.89	0.042	χ^2^(67) = 4,941.64***
M2 (Individual — full)	86,532	6	−56,083.10	112,306.21	112,961.99	0.043	χ^2^(2) = 40.64***
M3 (Individual + country)	65,864	10	−44,193.57	88,407.13	88,498.09	0.011	—

K = number of parameters (individual variables including the constant; country dummies are included in the model in M1 and M2 but are not shown in the K column). Log-LL = log-likelihood; LR = likelihood ratio test (compared with the previous model); McFadden *R*^2^ = McFadden pseudo-*R*^2^. M3 is a contextual effects model; it should not be directly compared within the same estimation framework as the fixed-effects models M1 and M2.

#### 4.3.2 Effects of individual-level variables (M2)

In M2, educational attainment emerges as the strongest individual predictor of environmental priority [OR = 1.242, 95% CI = (1.221, 1.263), *p* < 0.001]; this finding supports H1. A one-standard-deviation increase in educational attainment increases the odds of prioritizing the environment over the economy by approximately 24.2 percent. The gender variable indicates that women place greater importance on environmental priorities than men [OR = 1.075, 95% CI = (1.046, 1.110), *p* < 0.001]; this finding supports H2. Age exhibited a significant negative effect [OR = 0.950, 95% CI = (0.935, 0.965), *p* < 0.001] and indicates that an increase of one standard deviation in age (approximately 16.5 years) reduces the odds of prioritizing the environment over the economy by approximately 5.0 percent. This finding supports H3. Income has a significant negative effect [OR = 0.964, 95% CI = (0.950, 0.979), *p* < 0.001]; this finding supports H4. Place of residence also had a significant negative effect on M2 [OR = 0.933, 95% CI = (0.902, 0.965), *p* < 0.001]; urban residents reported a lower priority for the environment compared with rural residents. This finding also supports H5.

#### 4.3.3 Full model: combined effects of individual and country-level variables (M3)

Educational attainment remains a strong predictor of environmental priority when country-level variables are controlled for [OR = 1.189, 95% CI = (1.168, 1.221), *p* < 0.001]; this finding supports H1.

Among the country-level variables, GDP per capita has the strongest effect [OR = 1.246, 95% CI = (1.203, 1.290), *p* < 0.001]; this finding supports H6. Education expenditure as a proportion of GDP [OR = 1.130, 95% CI = (1.110, 1.150), *p* < 0.001] and the duration of compulsory education [OR = 1.056, 95% CI = (1.038, 1.074), *p* < 0.001] each exhibit significant positive effects independently; these findings support H8 and H7, respectively. CO₂ emissions are negatively associated with environmental priority [OR = 0.867, 95% CI = (0.838, 0.898), *p* < 0.001]; this finding supports H9. In M3, place of residence continued to show a significant negative effect [OR = 0.873, 95% CI = (0.842, 0.906), *p* < 0.001]; this finding also supports H5 in M3 (Table 5).

**Table 5:** M2 and M3 logistic regression results: Odds ratios and 95% confidence intervals.

Variable	*β*	SE	OR	95% CI lower	95% CI upper	*p*	Sig.
M2 — Individual-level model (*n* = 86,532)							
Education level (z-ISCED)	0.216	0.008	1.242	1.221	1.263	< 0.001	***
Gender (1 = Female)	0.073	0.014	1.075	1.046	1.110	< 0.001	***
Age (z)	−0.051	0.007	0.950	0.935	0.965	< 0.001	***
Income (z)	−0.036	0.007	0.964	0.950	0.979	< 0.001	***
Place of residence (1 = Urban)	−0.068	0.017	0.933	0.902	0.965	< 0.001	***
M3 — Full model (individual + country level) (*n* = 65,864)							
Education level (z-ISCED)	0.173	0.009	1.189	1.168	1.221	< 0.001	***
Gender (1 = Female)	0.065	0.016	1.067	1.038	1.097	< 0.001	***
Age (z)	−0.059	0.009	0.943	0.927	0.959	< 0.001	***
Income (z)	−0.038	0.008	0.963	0.947	0.978	< 0.001	***
Place of residence (1 = Urban)	−0.135	0.018	0.873	0.842	0.906	< 0.001	***
ln(GDP/capita) (z)	0.220	0.018	1.246	1.203	1.290	< 0.001	***
Duration of compulsory education (z)	0.054	0.009	1.056	1.038	1.074	< 0.001	***
Education expenditure % (z)	0.122	0.009	1.130	1.110	1.150	< 0.001	***
ln(CO₂/capita) (z)	−0.142	0.018	0.867	0.838	0.898	< 0.001	***

OR = odds ratio; CI = confidence interval. β and SE correspond to z-standardized variables; OR reflects the proportional effect on the odds of selecting an environmental priority of a one-standard-deviation increase in the relevant variable. Continuous variables are *z*-standardized (*M* = 0, SD = 1). M2: *n* = 86,532; M3: *n* = 65,864 (due to missing macro data). ***p < 0.001.

#### 4.3.4 Sensitivity analysis

To assess whether the individual-level estimates were sensitive to restricting the analysis to countries with complete country-level data, a sensitivity analysis was conducted. The M2 model, which contains only individual-level predictors, was re-estimated on the restricted analytic sample. The odds ratios obtained from this restricted sample were compared with those obtained from the full M2 sample. As Table 6 shows, the estimates are largely consistent across the two samples. The direction, statistical significance and approximate magnitude of all individual-level coefficients were preserved. Specifically, the odds ratios were nearly identical across the two samples: education (1.242 vs. 1.239), gender (1.075 vs. 1.075), age (0.950 vs. 0.959), income (0.964 vs. 0.960) and place of residence (0.933 vs. 0.923), with all coefficients remaining significant at *p* < 0.001 in both samples (see Table 6). Only marginal differences were observed for age and place of residence. This indicates that the individual-level results were largely unaffected by the country restriction. It does not, however, remove the limitation that the country-level findings are based on the 48 countries with complete contextual data.

**Table 6:** Sensitivity analysis: comparison of M2 odds ratios across the full and restricted samples.

Variable	M2 (full sample)	M2 (restricted subsample)
Education (*z*)	1.242	1.239
Gender (1 = female)	1.075	1.075
Age (z)	0.950	0.959
Income (z)	0.964	0.960
Place of residence (1 = urban)	0.933	0.923

Values are odds ratios. The full sample includes all respondents with complete individual-level data; the restricted subsample includes only respondents with complete country-level data (the same subsample used in M3). All coefficients remained statistically significant (*p* < 0.001) in both samples.

## 5 Discussion

This study aimed to examine the trade-off between environmental concern and economic growth across 66 countries using WVS 7 data, taking into account both individual and country-level factors. All nine hypotheses identified in the study were supported. However, the effect sizes indicate that country-level indicators are more influential than individual variables. This finding is consistent with Inglehart and Welzel’s (2005) approach based on self-expressive values. It also aligns with the findings of Franzen and Meyer (2010), who reported that environmental concern is higher in wealthier countries.

When the results are assessed in general terms, it is evident that the theory of post-materialism does not operate to the same extent at every level. Furthermore, the findings at the country level support the theory. However, the unexpectedly negative effect of income at the individual level calls into question the direct validity of the theory. This situation parallels the criticisms of the post-materialism thesis put forward by Brechin (1999) and Dunlap and York (2008). The findings also reveal that environmental concern cannot be explained by economic well-being alone. Factors such as the level of national well-being, the structure of the education system and economic dependence must also be taken into account. This study brings this multi-layered structure to light by addressing individual and country-level predictors within the same model. In this respect, the study contributes to research in comparative environmental sociology that examines individual and country-level factors together (Marquart-Pyatt, 2008; Gifford and Nilsson, 2014; Gelissen, 2007). The hypotheses are discussed in turn below.

### 5.1 H1: level of education and environmental priorities

The most significant finding of the study is that, across 66 countries and in all models, educational attainment is the strongest individual predictor of environmental prioritization. A one-standard-deviation increase in educational attainment increases the likelihood of prioritizing the environment over the economy by approximately 24 percent. This effect remains significant and maintains its impact even when controlling for country-level variables. This finding is consistent with Gifford and Nilsson’s (2014) assessment of the personal and social factors influencing environmental attitudes and behavior. The findings of Franzen and Vogl (2013), based on ISSP data covering 33 countries, also demonstrate that the relationship between education and environmental concern is positive and consistent. The present study confirms this finding with a broader range of countries and more recent data.

When country-level variables were included, the effect of education was found to diminish. This suggests that part of the individual effect of education may be related to variables such as country-level education expenditure and the duration of compulsory education. However, a mediation analysis is required to demonstrate this directly, and this falls outside the scope of our study. Education is not merely the transmission of information about the environment. It may also be associated with how individuals make sense of environmental issues and with their reported environmental sensitivity (Kollmuss and Agyeman, 2002; Stern, 2000). Similarly, Stern’s (2000) Values-Beliefs-Norms theory demonstrates how the relationship between values, beliefs and norms supports pro-environmental behavior. This theoretical framework helps to explain why education emerged as a strong individual predictor in the present study.

### 5.2 H2 and H3: gender and age

The finding that women exhibit a stronger environmental orientation than men is consistent with the comparative findings of Zelezny et al. (2000) across 14 countries. Gilligan’s (1982) framework of the ethics of care suggests that women’s greater emphasis on relationships, responsibility and protecting others may positively influence environmental attitudes. The findings of the present study, covering 66 countries, are also consistent with this explanation. However, these findings do not directly indicate the mechanism through which this difference arises. Xiao and McCright (2015) tested the hypothesis that the gender difference operates via organizational trust, but demonstrated that organizational trust does not explain this difference. Consequently, the literature suggests that explanations for the gender difference should not rely on a single mechanism.

Research findings indicate that young people prioritize the environment over the economy more frequently than older people. This finding is consistent with Inglehart’s (1977) cohort-based theory of post-materialism. Accordingly, generations raised during periods of greater economic security tend to gravitate more towards self-expression-oriented values such as environmental protection. Dunlap and York (2008) also present comparative findings showing that environmental concern is spreading across generations. The fact that the effect of age increases somewhat when country-level variables are included suggests that this difference may be particularly evident in countries with higher levels of education and higher GDP per capita. Therefore, it would be appropriate to test the interactions between age and country characteristics in future research.

### 5.3 H4: individual income level and environmental priorities

The negative association between individual income and environmental priority in both models is one of the notable findings of this study. However, because Q288 is a self-reported 10-point income scale, this result should be interpreted cautiously and should not be treated as direct evidence regarding the individual-level validity of post-materialist theory. This finding is partly at odds with the positive relationship demonstrated by Franzen and Vogl (2013) using ISSP data; however, it is consistent with the alternative forms of relationship highlighted by Brechin (1999) and Marquart-Pyatt (2008). Brechin (1999) demonstrated that environmental concern can be high in low-income countries, thereby emphasizing that the post-materialism thesis does not suggest that the income-environment relationship operates in a unidirectional and linear manner. Marquart-Pyatt (2008), on the other hand, found that the effect of individual income on environmental concern is relatively weak in industrialized countries and that this relationship can vary from country to country.

This contradiction indicates that individual income and national wealth operate through different mechanisms. In a multi-level context, the significance of individual income changes when differences in GDP between countries are controlled for. The scale reflects respondents’ reported income position rather than harmonized absolute income or purchasing power across countries. Future research may examine whether the relationship is nonlinear or varies according to national economic conditions.

### 5.4 H5: place of residence and urban–rural differentiation

The fact that the place of residence variable is negative and significant in both models indicates that urban individuals report lower environmental priorities than rural individuals. This result is partly consistent with common expectations in the literature. According to Gifford and Nilsson (2014), this relationship cannot be explained solely by reduced contact with nature; access to information, political ideology and value systems are also important. Consequently, this finding shows that urban living is not the sole factor directly suppressing environmental attitudes.

Particularly noteworthy is the fact that the effect of urbanization becomes stronger when country-level variables are included in the model. This finding suggests that the urban–rural association may also differ across national structural contexts. Urban residents may be more closely linked to consumption-oriented lifestyles and economic opportunities. They may also belong to groups that benefit from the industrial and service economy. These factors may contribute to a value hierarchy in which environmental concerns receive lower priority.

### 5.5 H6: *per capita* GDP and environmental prioritization

In this study, per capita GDP is the strongest country-level predictor of environmental prioritization. A one-standard-deviation increase in national prosperity increases the tendency to prioritize the environment over the economy by approximately 25 percent. This finding is directly consistent with Inglehart and Welzel’s (2005) theory of human development. In line with this theoretical account, higher national economic security is associated with stronger self-expression-oriented values. Individuals who have attained material prosperity at the national level tend to turn toward values such as environmental protection. This study also parallels the research by Franzen and Meyer (2010), which, using ISSP data, demonstrated the effect of cross-country GDP differences on environmental attitudes.

The positive association of GDP per capita with environmental priority is consistent with the contextual role of national economic security. However, it should not be directly contrasted with the individual income coefficient, because the two indicators capture different levels and forms of economic resources. The findings therefore suggest that the relationship between economic conditions and environmental priority may be context-dependent rather than uniform across levels of analysis. This finding points to the validity of the wealth hypothesis at the collective level. Dunlap and York (2008) note that such patterns require a reinterpretation of the post-materialism thesis rather than its rejection. Gelissen’s (2007) analysis also shows that, alongside economic development, the political system and institutional capacity influence environmental concern. This research therefore emphasizes the need to model GDP alongside indicators of structural education and environmental pressure.

### 5.6 H7 and H8: duration of compulsory education and education expenditure

The fact that the ratio of the duration of compulsory education and education expenditure to GDP exerts a significant positive effect on environmental priority, independent of individual-level variables, constitutes one of the original contributions of this study to the comparative environmental sociology literature. It is also noteworthy that the effect of education expenditure on environmental priority is approximately twice as strong as that of the duration of compulsory education. This difference is consistent with the possibility that characteristics of education systems, including public resources, are related to environmental priority beyond the duration of schooling. Marquart-Pyatt (2008) demonstrated the relationship between environmental concern and institutional capacity in industrialized countries, while Gelissen (2007) showed that country-level variables shape environmental concern. By introducing the ‘education system dimension’ into the literature, this study demonstrates that macro-level education indicators operate independently of individual education.

This finding suggests that environmental priority is positively associated not only with longer compulsory schooling but also with greater public investment in education. It may also reflect the place given to environmental literacy in education. The duration of compulsory education alone may therefore be insufficient; public investment may also be important. This conclusion points to a bridge that is rarely established between education policy and environmental policy. At the same time, it proposes a new analytical agenda for comparative education research from the perspective of environmental sociology.

### 5.7 H9: CO₂ emissions and environmental prioritization

The fact that CO₂ emissions show a significant negative correlation with environmental prioritization is one of the study’s contradictory yet striking findings. Contrary to expectations, comparative data indicate that environmental prioritization is lower in countries with high emissions. Dunlap and York (2008) interpreted this pattern as suggesting that, in economies dependent on fossil fuels, economic concerns may overshadow ecological awareness. In countries with high CO_2_ emissions and substantial industrial and energy production, people may be aware of environmental threats. However, the economic costs of environmental action may be difficult to bear.

When considered alongside Brechin’s (1999) findings, these results suggest that environmental priorities are not independent of the economic structure, but rather vary conditionally depending on the nature of that structure. Consequently, this study recommends that the variable ‘economic dependency structure’ be included in research on environmental priorities. From a comparative perspective, this finding is significant for climate policy and the energy transition. These patterns suggest that policy discussions in fossil-fuel-dependent economies may need to consider both economic justice and sustainable development when seeking public support. For example, environmental priority is 68.5 percent in Iran, compared with 36.6 percent in Egypt and Tunisia. This difference of approximately 32 percentage points suggests that environmental priorities may be related not only to culture but also to country-specific economic dependency structures and CO_2_ intensity (Marquart-Pyatt, 2008).

The modest explanatory power reported for the models should not be seen as a weakness specific to this study. Similar WVS- and ISSP-based studies on environmental attitudes report comparably modest explanatory power, since socio-demographic predictors alone can only partially account for a complex construct like environmental prioritization (Marquart-Pyatt, 2008; Franzen and Vogl, 2013). Other factors outside standard socio-demographic models-such as perceived risk, political ideology, and institutional trust-are also known to shape environmental attitudes (Gifford and Nilsson, 2014). For this reason, the practical significance of the findings should be judged by the direction and consistency of the effects rather than by the amount of variance explained (Hosmer et al., 2013).

### 5.8 Limitations and future research

The findings of this study should be interpreted in light of several limitations. Each limitation also points to a direction that future research could take.

This study is based on cross-sectional data and therefore does not establish temporal ordering or causal effects. The findings should be interpreted as associations rather than causal relationships, since reverse causation and unmeasured confounding cannot be ruled out. Future research could address this by using panel analyses across successive waves of the WVS. Value change could then be traced over time, and causal relationships could be tested more directly.

Item Q111, the dependent variable, measures the priority given to environmental protection versus economic growth using a single dichotomous question. Although this measure does not reflect all dimensions of environmental attitudes, it is widely used in large-scale comparative studies. Later studies could complement this item with multi-item measures that capture the different dimensions of environmental attitudes more fully.

Due to missing country-level indicators, M3 was estimated for 48 countries, which limits the generalizability of the country-level findings. The exclusion of countries with missing contextual indicators may introduce selection bias; future research should use datasets with more complete macro-level coverage.

Individual income was measured using a self-reported 10-point scale and may not represent equivalent levels of absolute income or purchasing power across countries. The study also did not test whether the association between income and environmental priority is nonlinear or varies across national economic contexts. Future research could examine these possibilities using polynomial or spline terms and theoretically specified cross-level interactions.

Although the WVS sampling design is highly representative at the national level, the quality of implementation may vary between countries. This may limit the validity of cross-national comparisons. Researchers could take this into account by using indicators of data quality, or by cross-checking the results against other cross-national datasets.

Cross-cultural measurement invariance has not been systematically tested in this study. It should therefore be borne in mind that item Q111 may have been interpreted differently in various cultural contexts. Testing measurement invariance across the countries included in Wave 7 of the WVS would help to establish whether the measure is comparable, and this is an important next step. Cross-level interactions were also not examined. Future research using theoretically specified moderation hypotheses and broader country coverage could investigate whether the association between individual education and environmental priority varies with national education expenditure.

Beyond these specific points, the model used here could be extended in several ways. One would be to include cultural region as a categorical variable, so that regional effects can be tested directly while within-region heterogeneity is taken into account. Another would be to estimate cross-level interaction models, such as age × GDP, in order to examine how individual and contextual factors operate together. A third would be to test formally whether individual education mediates the association between the structural characteristics of the education system and environmental priority. Taken together with panel analyses across successive WVS waves, these extensions would show more clearly how robust the present findings are, and how environmental values change within the education system.

## 6 Conclusion

This study has examined the individual- and country-level determinants of individuals’ preferences between environmental protection and economic growth from an international comparative perspective. A total of nine hypotheses-five at the individual level and four at the country level-were tested; all hypotheses were statistically supported. The findings revealed that, at the individual level, education is the strongest determinant of environmental priority; that women exhibit a stronger environmental orientation than men; and that environmental priority decreases with age. Conversely, individual income had a negative effect, and it was observed that individuals living in urban areas have a lower environmental priority compared to those in rural areas. At the national level, GDP per capita emerged as the strongest macro-level variable, while the duration of compulsory education and expenditure on education showed a positive relationship, and CO₂ emissions showed a negative relationship.

When these findings are considered together, it appears that country-level economic indicators and the structural characteristics of the education system have a stronger influence on the importance attached to the environment than individual factors. This result partially supports the theory of post-materialism (Inglehart and Welzel, 2005). However, while the post-materialist shift in values appears consistent at the national level, the negative effect of individual income-contrary to expectations-suggests that this theory should be interpreted not as operating directly at the individual level, but rather as a common mechanism that operates through the general level of societal wellbeing.

The findings also have implications for the post-materialist account of environmental priority. The positive association between GDP per capita and environmental priority is consistent with the view that economic security at the country level is associated with stronger self-expression values. However, the negative association found for self-reported individual income shows that this relationship does not operate in the same way at the individual and country levels. The positive associations of compulsory education duration and education expenditure further indicate that environmental priority is related not only to individuals’ education but also to the broader institutional characteristics of national education systems.

These findings point to three key policy implications. Firstly, extending the duration of compulsory education is not sufficient in itself. This is because the level and quality of public resources allocated to education are also decisive factors in the development of environmental values. This highlights the importance of education systems that prioritize environmental literacy and are supported by high-quality public investment. Secondly, the low priority given to environmental issues in high-emission economies demonstrates that environmental policies cannot be based solely on raising awareness. Such policies must also take into account economic dependencies and livelihood conditions. Thirdly, the widespread establishment of economic security at the national level emerges as a key prerequisite for supporting the transformation of environmental values.

The original contribution of this study lies in its presentation of a comprehensive comparative analysis that examines both individual and country-level variables using data from 66 countries in the 7th wave of the World Values Survey (WVS). The study also empirically demonstrates that the structural characteristics of the education system-namely, the duration of compulsory education and expenditure on education-predict environmental priority independently of the effect of individual education. This finding highlights the need to consider the relationship between education and the environment not only at the individual level but also at the structural and institutional levels, and opens up a new analytical field at the intersection of comparative environmental sociology and education research.

## Data Availability

Publicly available datasets were analyzed in this study. The data analyzed in this study are publicly available at www.worldvaluessurvey.org. The WVS Wave 7 dataset (Version 6.0.0) can be downloaded free of charge.
